# Paclitaxel-induced lung injury and its amelioration by parecoxib sodium

**DOI:** 10.1038/srep12977

**Published:** 2015-08-10

**Authors:** Wen-jie Liu, Zhong-jian Zhong, Long-hui Cao, Hui-ting Li, Tian-hua Zhang, Wen-qian Lin

**Affiliations:** 1 Sun Yat-Sen University Cancer Center, State Key Laboratory of Oncology in South China, Collaborative Innovation Center of Cancer Medicine, Guangzhou, Guangdong, PR China.; 2 Department of Anaesthesia, Sun Yat-Sen University Cancer Center, Guangzhou, Guangdong, PR China.; 3 Department of Blood transfusion, Sun Yat-Sen University Cancer Center, Guangzhou, Guangdong, PR China.; 4 Pain management of the first affiliated hospital of Zheng-Zhou University, Zhengzhou, Henan, PR China.

## Abstract

To investigate the mechanism of paclitaxel-induced lung injury and its amelioration by parecoxib sodium. In this study, rats were randomly divided into: the control group (Con); the paclitaxel chemotherapy group (Pac); the paclitaxel+ parecoxib sodium intervention group (Pac + Pare); and the parecoxib sodium group (Pare). We observed changes in alveolar ventilation function, alveolar-capillary membrane permeability, lung tissue pathology and measured the levels of inflammatory cytokines and cyclooxygenase-2 (Cox-2) in lung tissue, the expression of tight junction proteins (Zo-1 and Claudin-4). Compared with the Con group, the lung tissue of the Pac group showed significantly increased expression of Cox-2 protein (p < 0.01), significant lung tissue inflammatory changes, significantly increased expression of inflammatory cytokines, decreased expression of Zo-1 and Claudin-4 proteins (p < 0.01), increased alveolar-capillary membrane permeability (p < 0.01), and reduced ventilation function (p < 0.01). Notably, in Pac + Pare group, intraperitoneal injection of parecoxib sodium led to decreased Cox-2 and ICAM-1 levels and reduced inflammatory responses, the recovered expression of Zo-1 and Claudin-4, reduced level of indicators reflecting the high permeability state, and close-to-normal levels of ventilation function. Intervention by the Cox-2-specific inhibitor parecoxib sodium can block this damage.

Neoadjuvant chemotherapy is the primary preoperative treatment for patients with various malignant solid tumors. It has been reported that after neoadjuvant chemotherapy, pulmonary function tests show a reduced carbon monoxide diffusing capacity, and the incidence of postoperative pulmonary complications is also significantly increased^1^,^2^, suggesting that neoadjuvant chemotherapy may induce lung injury. Alveolar diffusion relies on the normal function of the alveolar-capillary barrier, and tight junctions (TJs) between epithelial cells and endothelial cells are an important component of the alveolar-capillary barrier. TJs are composed of the occludin, zonula occludens (Zo), and claudin protein families. Changes in these proteins may undermine the TJ integrity, open the cellular junctions, and lead to an increased permeability of the alveolar-capillary membrane (ACM) and congregation of high-protein liquids and cells to the lung interstitium and the alveolar cavities, resulting in pulmonary diffusion dysfunction^3^,^4^,^5^,^6^. Paclitaxel is a commonly used neoadjuvant chemotherapy drug. Previous studies have found that multi-course paclitaxel chemotherapy can cause lung injuries such as pulmonary alveolar-capillary leakage. It has not been reported whether neoadjuvant chemotherapy damages TJs, in turn resulting in lung injury. In addition, it has been reported that paclitaxel can cause the abnormal expression of cyclooxygenase-2 (Cox-2) in lung tissue^7^,^8^,^9^. Cox-2 is a pro-inflammatory factor and may cause lung injury by activating a cascade of inflammatory reactions, which might be the mechanism of paclitaxel-induced lung injuries. Whether the application of specific Cox-2 inhibitors can block this type of lung injury remains to be investigated.Therefore, utilized a rat model of single-course paclitaxel chemotherapy, the primary objective of this study was to clarify the characteristics and mechanisms of paclitaxel chemotherapy-induced lung injury by detecting the alveolar-capillary barrier permeability, the expression of lung tissue inflammatory cytokines, and the levels of Cox-2 and TJ proteins (Zo-1 and Claudin-4). Furthermore, the overarching goal of this study was to explorelung injury amelioration by the Cox-2-specific inhibitor parecoxib sodium, thus providing experimental evidence for the prevention and treatment of postoperative pulmonary complications in patients receiving neoadjuvant chemotherapy.

## Results

### Inter-group comparison of arterial blood gas parameters

**Figure 1:**
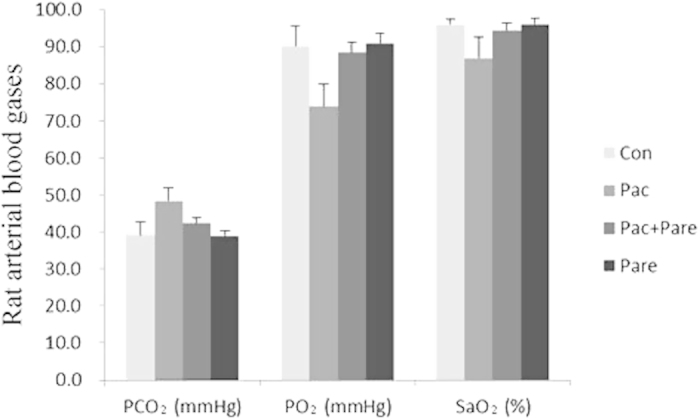
The effect of different treatments on rat arterial blood gas values. The PCO_2_ of the Pac group was significantly higher compared with the Con group, while PO_2_ and SaO_2_ levels significantly decreased, p < 0.01; there were no significant differences among the Con group, the Pac + Pare group, and the Pare group in the three indicators. The data are presented as x ± s.

Compared with the other groups, the Pac group showed significantly increased arterial pressure of carbon dioxide (PCO_2_) levels and significantly decreased partial pressure of oxygen (PO_2_) and arterial oxygen saturation (SaO_2_) levels (p < 0.01). The Pac + Pare group, the Pare group, and the Con group showed no significant differences in the above parameters (p > 0.05), as shown in Fig. 1.

### Histological observation of lung tissues from different groups

**Figure 2:**
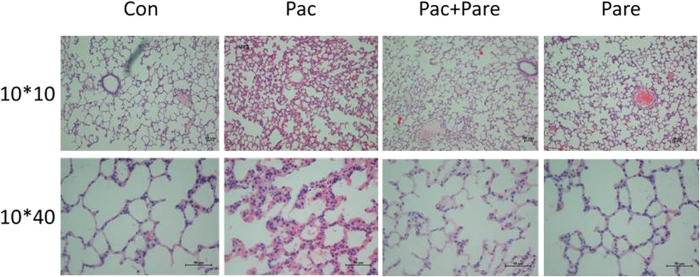
Histological observations of the four groups of lung tissues (HE staining). The Con group was normal lung tissue. The structure of the pulmonary alveoli was clear, and the alveolar wall was thin. The Pac group showed severe inflammation, the alveolar structures were disordered, and there were edema and broadening in the lung interstitium and hemorrhage in both the alveolar cavity and the interstitium. The Pac + Pare group showed significantly reduced symptoms compared with the Pac group, with only slight edema. The Pare group was more similar to the Con group.

The Con group showed clear and intact lung tissue structures, showing no inflammatory cell infiltration, bleeding, or edema or thickening of the alveolar wall. The Pac group showed disorganized alveolar structure, significant exudation of inflammatory cells, red blood cells scattered in multiple alveolar cavities, and pulmonary interstitial edema and thickening. The pathological damage was significantly milder in the Pac + Pare group than in the Pac group, with clear alveolar structures, insignificant edema and thickening, and significantly reduced inflammation. The pathological findings of the Pare group were similar to those of the Con group (Fig. 2). The Pac group was significantly different from the other three groups in pathology damage score (p < 0.01), while there were no significant differences among the Con group, the Pac + Pare group, and the Pare group (Table 1).

### Comparison of cell counts in the broncho-alveolar lavage fluid (BALF) of various groups

**Figure 3:**
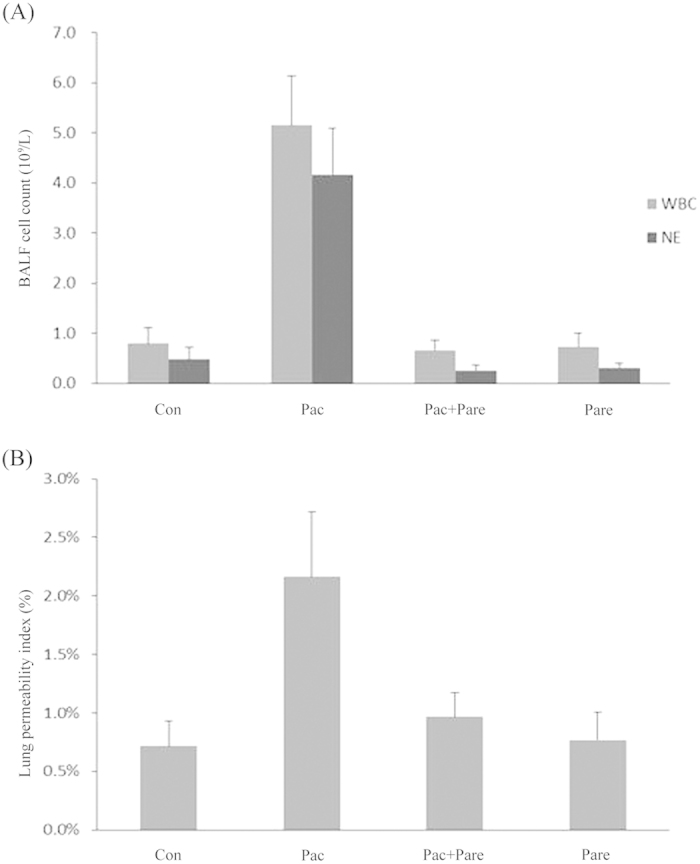
(**A**) White blood cell (WBC) and neutrophils (NE) counts in BALF: the total number of WBCs was sharply increased in the Pac group, which was dominated by NEs. In other three groups, there were fewer cells, and NE occupied a smaller percentage; p < 0.01. The data were presented as x ± s; (**B**) The lung permeability index (LPI, BALF protein/serum protein) change for the four different groups: the LPI was significantly increased in the Pac group (2.2 ± 0.56%) compared with the Con group (0.72 ± 0.22%), the Pac + Pare group (0.97 ± 0.21%), and the Pare group (0.77 ± 0.24%), p < 0.01. The data are presented as x ± s.

The categorized counting results of total white blood cells (WBCs) and neutrophils (NEs) in BALF showed that the cell number of the Pac group was significantly higher than the other three groups (p < 0.001), mainly consisting of NE increases. There were no significant differences among the Pac + Pare group, and the Con group (p > 0.05), as shown in Fig. 3(A).

### Comparison of the lung permeability index (LPI) among groups

The four groups had BALF recovery rates above 87%, and there were no significant differences among groups. The LPI (2.2 ± 0.56%) of the Pac group was significantly higher than that of the other three groups (p < 0.01). The LPI of Con group, the Pac + Pare groupand the Pare group were as follow, 0.72 ± 0.22%, 0.97 ± 0.21%, 0.77 ± 0.24% [p > 0.05; Fig. 3(B)].

### Comparison of the Evans blue contents of lung tissues from different groups

**Figure 4:**
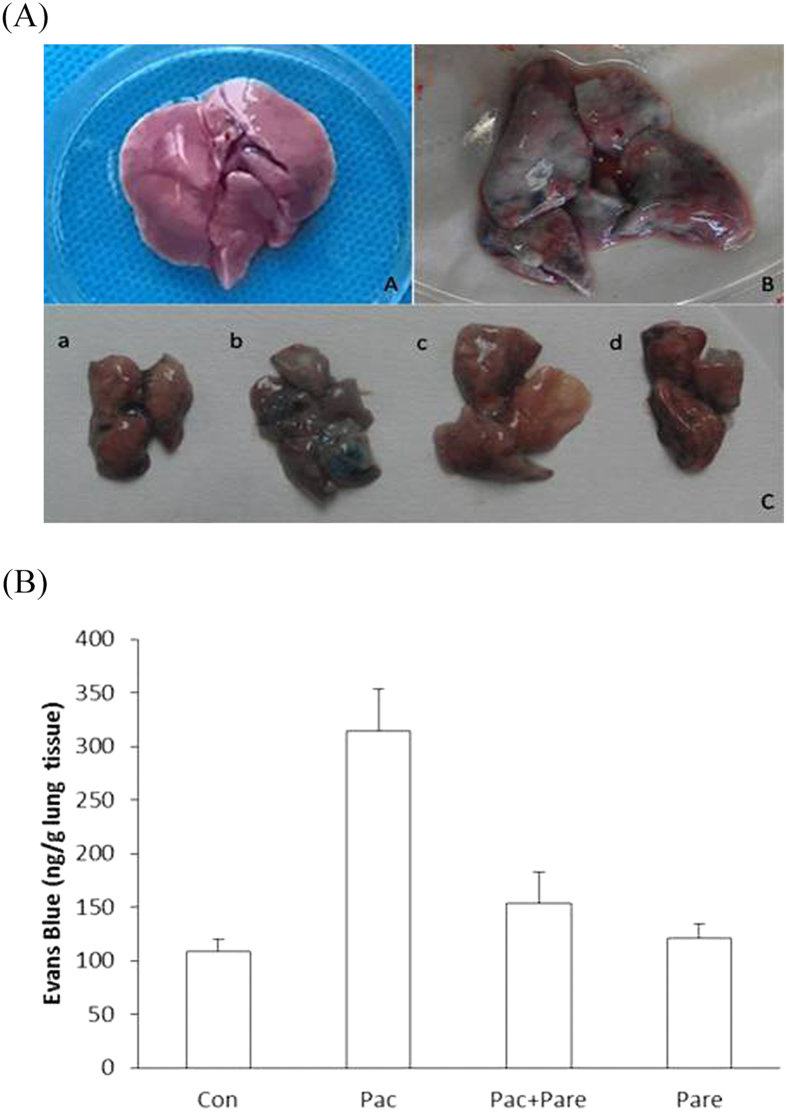
(**A**) Normal lung and lung tissue stained with Evans Blue: A. Normal lung tissue; B. Pac group lung stained with Evans blue; C. a, b, c, d were lung tissues collected from the Con, Pac, Pac + Pare, and Pare group of rats, respectively, 30 min after Evans blue iv.; (**B**) Content of Evans blue per gram of lung tissue: evans blue in the Con group was 108 ± 12.1 ng/g and 314 ± 38.7 ng/g in the Pac group, p < 0.01; there were no significant differences among the Con group, the Pac + Pare group (154 ± 28.6 ng/g), and the Pare group (120 ± 13.5 ng/g). The data are presented as x ± s.

It was macroscopically obvious that the lung tissue of the Pac group stained a more intense blue than the other three groups [Fig. 4(A)]. The test results showed that the Evans blue content of the Con group was 108 ± 12.1 ng/g lung tissue, and those of the Pac group, the Pac + Pare group, and the Pare group were 314.3 ± 38.7 ng/g lung tissue, 154 ± 28.6 ng/g lung tissue, and 120.7 ± 13.5 ng/g lung tissue, respectively. The Evans blue content of the Pac group was significantly higher than of the other three groups [p < 0.01; Fig. 4(B)].

### Western blot detection of Cox-2, intercellular adhesion molecule-1 (ICAM-1), Zo-1, and Claudin-4 proteins

**Figure 5:**
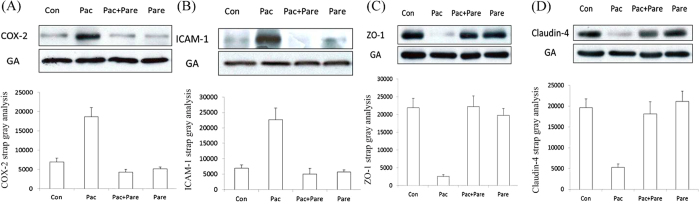
Cox-2, ICAM-1, Zo-1, and Claudin-4 protein expression levels in rat lung tissue after the different treatments in each group. Immunoblotting showed increases in Cox-2 and ICAM-1 and a reduction in Zo-1 and Claudin-4 after one course of paclitaxel compared with normal lung tissue; p < 0.01. These changes were reversed by using parecoxib in combination with paclitaxel, while the group that received parecoxib treatment alone resembled the Con group. The bar graph under the protein strap represents the corresponding band intensities; the data are presented as x ± s and corrected by GA.

The Con group had very low expression levels of Cox-2 and ICAM-1 and normal expression levels of Zo-1 and Claudin-4. The Pac group had significantly increased Cox-2 and ICAM-1 levels, while Zo-1 and Claudin-4 expression levels were significantly reduced. The Pac + Pare had normal Cox-2 and ICAM-1 expression levels and recovered Zo-1 and Claudin-4 expression, differing from the Pac group. (Fig. 5).

## Discussion

In this study, we used a daily single course of 2 mg/kg/d paclitaxel via intraperitoneal injection in rats to simulate lung injuries and pathophysiological changes in patients after neoadjuvant chemotherapy. Experimental results showed the following: (1) the pathological sections of the Pac group showed edema, exudation, and thickening of the alveolar septa, inflammatory cell infiltration, and small patches of red blood cells in alveolar cavity, suggesting that the inflammatory response was involved in the injury process and confirming the increased water and cell permeability of the ACM; (2) the Pac group of lung tissue showed increased Evans blue content and LPI. These two results suggested that the physical barrier and electrochemical barrier functions of ACM were damaged, leading to an increased permeability for macromolecules and charged molecules; (3) blood gas analysis results showed that the Pac group had increased PCO_2_ levels and decreased PO_2_ and SaO_2_ levels, which were significantly different from those of the Con group, indicating that paclitaxel damaged the ACM and thereby affected gas exchange. These three groups of indicators all reflected ACM permeability, with consistent trends of changes, and, from three aspects, demonstrated the impaired ACM barrier function after paclitaxel application: significantly increased permeability; the inability to regulate substance transmembrane transportation; and the free infiltration of water, proteins, cells, and Evans blue from capillaries to alveolar interstitium and alveolar cavity, causing ACM thickening and slowed CO_2_ and O_2_ diffusion.

Pulmonary ventilation function (normal diffusion of CO_2_ and O_2_) relies on the separation of the alveolar cavity and pulmonary capillary lumen and the maintenance of ACM thickness within a certain range, which depend on the intact function of the alveolar-capillary barrier. TJ is a complex formed by the membrane proteins located on the apical surface of the sidewalls of adjacent epithelial/endothelial cells. TJ couples with the cytoskeleton inside cells to regulate the transportation of water, ions, and large molecules via the paracellular pathway and is an important structure protecting the alveolar-capillary barrier function. It is mainly composed of the occludin, zo, and claudin protein families. Zo-1 and Claudin-4 are important components of TJ, and the reduction in their expression levels has been proven to play a key role in the injury and increased permeability of a variety of epithelial/endothelial cells^3^,^9^,^10^. In this study, after paclitaxel application, the Pac group showed reduced expressions of the TJ proteins Zo-1 and Claudin-4, which is consistent with the damaged ACM barrier, the increased permeability, and the CO_2_ and O_2_ diffusion disorders observed in this group. These results for the first time confirmed that paclitaxel-induced ACM damage is related to its effect on TJs. These data are consistent with previous studies showing that TJs play an important role in maintaining the alveolar-capillary barrier, as suggested by TJ Mitchell LA, *et al*.^3^, and Claudin-4 can protect ACM permeability changes in acute lung injury and lung injury during the mechanical ventilation, as indicated by Wray C, *et al*.^4^. We believe that paclitaxel inhibits the expression of Zo-1 and Claudin-4 and affects TJ integrity, resulting in alveolar-capillary barrier dysfunctions, increased ACM permeability, and exudation of ferrihemoglobin fluid and cells from the capillaries to the alveoli interstitium and even the alveolar cavity, which causes pulmonary edema and, ultimately, ACM thickening and gas diffusion dysfunctions^5^,^6^.

In this study, paclitaxel-induced reductions in the expression of TJ components were consistent with the paclitaxel-induced enhancement of Cox-2 levels in the tissue, suggesting that high Cox-2 expression may be involved and play an important role in the process of paclitaxel-induced TJ injury. Cox-2 is an inducible cyclooxygenase and can be produced by a variety of cells including neutrophils, and it can inhibit neutrophil apoptosis. Cox-2 and neutrophils cooperate to cause an amplified and enhanced inflammatory response and generate cytotoxic effects, which are important in the lung injury^11^,^12^,^13^,^14^. ICAM-1 can be induced by inflammatory mediators in a variety of structural cells. It is involved in the adherence between neutrophils and structure cells and is critical for neutrophil functions^15^,^16^. Studies on paclitaxel drug resistance have confirmed that paclitaxel induces the expression of Cox-2 mRNA and increased Cox-2 levels in epithelial and endothelial cells^7^,^8^, which may be because paclitaxel stimulates Cox-2 transcription and enhances Cox-2 mRNA stability^17^. In this study, the pathological sections of the rats in the Pac group showed inflammation, neutrophil infiltration, a significantly increased number and ratio of NEs in BALF, and significantly increased Cox-2 and ICAM-1 in tissue homogenates. These indicators are manifestations of pulmonary inflammations, and the results confirmed that paclitaxel enhanced Cox-2 expression in lung tissues and triggered or magnified inflammation. Studies on TJ functions in a variety of tissues often found that reduced TJ function and reduced protein expression were parallel to Cox-2 high expression^18^,^19^,^20^; however, current research studies have not confirmed the existence of a causal relationship between them. In this study, application of the specific Cox-2 inhibitor parecoxib sodium led to decreased Cox-2 and ICAM-1 levels in lung tissues and reduced inflammatory responses as indicated by the pathological sections and BALF, the recovered expression of the TJ proteins Zo-1 and Claudin-4, reduced levels of various indicators reflecting the high permeability state of lung tissue, and close-to-normal levels of indicators of pulmonary ventilation function such as PCO_2_, PO_2_ and SaO_2_. This study confirmed that paclitaxel-induced lung injuries result from inducing Cox-2 high expression, triggering/magnifying inflammation, and affecting proteins. As well as, the application of the Cox-2 inhibitor parecoxib sodium reversed the ACM damages caused by paclitaxel, which have not been reported previously.

Previously, Mirzapoiazova *et al*. have revealed that intravenously delivered taxol significantly reduced inflammatory histological changes in lung parenchyma and paremeters of lipopolysaccharide (LPS)-induced inflammation. However, we failed to show the same results. We believed that can be explained by two particular factors. First, the different model of murine lung injury may lead to this phenomenon. Mirzapoiazova *et al*. use aninflammatory model induced by intratracheal LPS-induced administration. In the present study, the Pac group was induced by intraperitoneal injection of paclitaxel. Second, heterogeneous of the parameters we detected may also lead to the bias. In addition, intricate mechanisms of inflammation induced by various agents also affect our results. Therefore, this conclusion merits additional research^21^.

Neoadjuvant chemotherapy often increases the surgical risk, causing postoperative pulmonary complications. Studies have found that diffusing capacity of the lung for carbon monoxide (Dlco%) and Dlco/the alveolar volume (Va%) reduction detected by preoperative pulmonary function tests may indicate surgical risks and predict complications; the mechanisms are not yet clear, but they may relate to pneumonia and an increased lung permeability^1^,^2^; postponing surgery until the Dlco% and Dlco/Va% values have recovered may reduce surgical risks^1^. The commonly used chemotherapy drug paclitaxel can affect lung functions, especially Dlco^22^. Dlco is the diffusing capacity of the lung for carbon monoxide and is the gold standard reflecting ACM ventilation functions. ACM thickening and fibrosis due to various causes can lead to reduced Dlco. In this study, we demonstrated that paclitaxel destroyed the alveolar-capillary barrier, leading to ACM exudation and thickening, thus affecting gas diffusion; the specific mechanism is the Cox-2-mediated inflammatory response with neutrophil involvement, and the specific Cox-2 inhibitor can antagonize paclitaxel-induced TJ decrease and ACM damage.

Diffuse ACM injury and postoperative pulmonary complications caused by paclitaxel neoadjuvant chemotherapy are widely present in clinical practices. These injuries and complications are related to the treatment courses and accumulated dose, but there are currently no effective treatments. Parecoxib sodium is effective for the treatment of other types of lung injuries, such as acute lung injury caused by multi-organ failure, burns, and pancreatitis^11^,^12^,^13^. However, the treatment of paclitaxel-induced lung injuries using parecoxib sodium was limited. This study provides a option, which was innovative, combining neoadjuvant chemotherapy with Cox-2 inhibitors may reduce inflammatory histological changes in lung parenchyma and ameliorate the parameters of inflammatory response, permeability state, and ventilation function.

Paclitaxel-induced increases in Cox-2 are related to the occurrence of drugresistance. Cox-2 inhibitors can significantly reduce paclitaxel drug resistance and enhance its anti-tumor sensitivity. Clinical studies have shown that Cox-2 can promote angiogenesis and accelerate tumor growth and metastasis^23^,^24^, and it can even serve as a prognostic marker for a variety of tumors. Clinical trials have shown that paclitaxel combined with anti-Cox-2 treatment can achieve good anti-tumor effects^7^,^23^,^25^,^26^.

In conclusion, our study show that paclitaxel neoadjuvant chemotherapy can induce ACM injury and Cox-2 inhibitors attenuates this damage, providing further evidence to promote the regimens of preoperative chemotherapy combined with Cox-2 inhibitors.

## Methods

Study protocol was approved by the Ethics Committee of Sun Yat-Sen University Cancer Center. Furthermore, all animal experiments were performed in accordance with guidelines of Institutional Animal Care and Use Committee of Sun Yat-Sen University.

### Animals and Groups

A total of 28 healthy female Wistar rats, approximately 9weeks of age and weighing 200-220 g, provided by the Sun Yat-sen University South campus Experimental Animal Center, were used for the study. The rats were housed in the specific pathogen-free environments , had free access to food and water ad libitum and were maintained on a 12-hour light-dark cycle. The rats were randomly assigned to four groups with 7 rats in each group: (1) control group (Con); (2) paclitaxel chemotherapy group (Pac); (3) paclitaxel+ parecoxib sodium intervention group (Pac + Pare); (4) parecoxib sodium group (Pare). In accordance with the methods reported in the literature, the rats in the Pac group received a daily intraperitoneal injection of 0.5 mg/ml paclitaxel (Taxol^®^, Bristol Myers Squibb, SRL) at a dose of 2 mg/kg for six consecutive days; those in the Con group received a daily intraperitoneal injection of 1 ml of normal saline (NS) for six consecutive days. The rats in the Pac + Pare group received a daily intraperitoneal injection of paclitaxel at a dose of 2 mg/kg for six consecutive days and also received intraperitoneal injections of parecoxib sodium (Dynastat^®^, Pfizer Limited, Sandwich, UK) at a dose of 2 mg/kg twice per day (one in the morning and one in the evening) from the fourth day to the sixth day; those in the Pare group received a daily intraperitoneal injection of saline at 4 ml/kg during the first four days and then received intraperitoneal injections of parecoxib sodium at a dose of 2 mg/kg twice per day (one in the morning and one in the evening) from the fourth day to the sixth day. Normal food and water were provided. The rats were killed by bleeding under anesthesia on the eighth day after drug administration.

### Arterial blood gas analysis

The rats received 10% chloral hydrate (3 ml/kg) by 12 intraperitonealinjection and were stabilized; 2 ml of blood was collected from the right carotid artery. A small portion of the blood was collected for blood gas analysis (blood Gas Analyzer) (Abbott, USA); the remaining blood was centrifuged (3000 r/min, r = 15 cm), and the protein concentration in the supernatant was measured using the bicinchoninic acid (BCA) method.

### Lung tissue collection and pathology scoring

After the rats were sacrificed, the chest was opened to dissect the right lung. The right middle lobe of the lung was fixed in 40 g/L paraformaldehyde and processed into paraffin sections. Slices were stained using hematoxylin and eosin (HE) and observed under a light microscope. A 4-point scoring method was used to evaluate the inflammatory changes of the lung tissue, hemorrhage in the alveolar cavity, and edema and thickening of the alveolar wall: 0 represents no pathological changes; 1 indicates mild changes in an area of 30% or less; 2 indicates moderate changes of 30–60%; and 3 indicates severe changes of more than 60%. The remaining right lung was frozen in a liquid nitrogen tank for future tissue examination.

### Alveolar perfusion, cell count, and lung permeability index measurement

The right main bronchus was ligated to perform alveolar perfusion of the left lung. A tracheotomy was performed, and a 12th needle was inserted into the trachea, sutured, ligated, and fixed to prevent overflow of BALF. A total of 5 ml of sterile NS at 4 °C was slowly injected through the catheter in three installments, followed by repeated aspirations; the total amount of recovered fluid was above 3.8 ml. The animals were placed in the Trendelenburg position at the end. The BALF was placed in sterile centrifuge tubes and centrifuged (1500 r/min, r = 15 cm, 10 min, 4 °C). The protein concentration in the BALF supernatant was determined using the bicinchoninic acid (BCA) method, and the lung permeability index (LPI) was calculated (BALF protein concentration/serum protein concentration). The precipitated cells were washed and centrifuged twice in NS, then resuspended with 1 ml of NS and dispersed into cell suspensions by pipetting and mixing to perform cell counting in the Neubauer cell counting chamber. A smear of sediment cells was stained with HE; at least 200 cells were counted for cell classification.

### Measurement of lung permeability using Evans blue

Arterial blood was collected from three rats in each group, and the rats were injected with 6 mg/ml Evans blue (30 mg/kg) (Evans blue and formamide (Amresco, USA) via the jugular vein. After 30 min, the chest was opened, the right atrium was cut, and the pulmonary circulation was flushed with 100 ml of NS via the right ventricle at a pressure of 20 cm H2O. The two lungs were removed, the trachea was cut, and the lung tissue was cleaned, dried, weighed, and homogenized. The lung homogenate was soaked in formamide at 1 ml/100 mg lung tissue and incubated 18 h at 60 °C, followed by centrifugation at 12000 r/min (r = 6 cm) for 20 min. The supernatant was measured at 620 nm to obtain the absorbance (a Unicam Heliox UV-visible spectrophotometer, UK) and the lung tissue formamide content was calculated in accordance with the formamide standard curve.

### Detection of the protein expression of Cox-2, ICAM, Zo-1, and Claudin-4 in lung tissues

Frozen lung tissue was collected and rinsed with ice-cold saline to remove the blood. The tissue was then dried with filter paper. A 100-mg portion of lung tissue was placed in a mortar and shredded with ophthalmic scissors as quickly as possible. Liquid nitrogen was then added to the mortar, the tissue was ground to a paste, and 1 ml of protein lysis buffer was added. The lysate was placed on ice for 20 min, followed by centrifugation at 12000 r/min at 4 °C for 30 min. The supernatant was centrifuged for 15 min. According to the kit manuals, Western blot assays were used to detect the protein expression levels of Cox-2 (anti-Cox-2 polyclonal goat antibody, 3362-100, Biovision, USA), ICAM-1 (anti-ICAM monoclonal mouse antibody, NB500-31, Novus Biologicals, USA), Zo-1 (anti-Zo-1 polyclonal rabbit antibody, 617300, Invitrogen, USA), and Claudin-4 (anti-Claudin-4 monoclonal mouse antibody, 329400, Invitrogen, USA) in lung tissue (a Bio-Rad 550 microplate reader, USA).

### Statistical analysis

Data are expressed as mean ± SEM(x ± s.). Comparison of the means of multiple groups of samples was conducted using analysis of variance, and comparison of the means of two sample groups was conducted using t tests. A P value of less than 0.05 was considered statistically significant. All analyses were conducted using statistical software package, version 13.0 (SPSS Inc., Chicago, IL).

## Additional Information

**How to cite this article**: Liu, W.-J. *et al*. Paclitaxel-induced lung injury and its amelioration by parecoxib sodium. *Sci. Rep.* **5**, 12977; doi: 10.1038/srep12977 (2015).

## Figures and Tables

**Table 1:** Pathological damage score of lung in each group.

	Con	Pac	Pac + Pare	Pare
Score	0.5 ± 0.15	2.6 ± 0.29^a^	0.7 ± 0.25^b^	0.6 ± 0.14^b^

^a^Compared with the other groups, p < 0.01.

^b^Compared with Con group, there was no significant different; Pare group was no difference with Pac + Pare group. Data were presented as 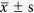.
